# Determining the Vapour Resistance of Breather Membrane Adhesive Joints

**DOI:** 10.3390/ma15196619

**Published:** 2022-09-23

**Authors:** Fride Engesland Fuglestad, Erlend Andenæs, Stig Geving, Tore Kvande

**Affiliations:** 1 Department of Civil and Environmental Engineering, Norwegian University of Science and Technology, 7491 Trondheim, Norway; 2 SINTEF Community, 7465 Trondheim, Norway

**Keywords:** breather membrane, vapour resistance, adhesive joints, mould growth

## Abstract

Due to increasingly stringent requirements, tapes and adhesive joints are a commonly used method to ensure tightness and energy efficiency in modern building envelopes. Previous studies have researched and tested properties such as the strength and tightness of adhesive joints. So far, water vapour resistance has been neglected. This article aims to determine the vapour resistance and shed light on possible consequences of vapour-tight adhesive joints in breather membranes used in roof assemblies. Laboratory measurements of vapour resistance were conducted according to NS-EN ISO 12572:2016, known as the cup method. Eleven products of breather membranes were tested. Results from the laboratory measurements were used to evaluate the impact of vapour-resistant adhesive joints related to the drying of built-in moisture. The simulation programs WUFI 2D and WUFI Mould Index VTT were used to model scenarios for moisture transport and risk for mould growth. Laboratory results show that the vapour resistance of breather membrane adhesive joints varies from 1.1 to 32 m in s_d_-value. Three of the tested products have a vapour resistance larger than 10 m, while four products have an s_d_-value less than 2.0 m. The s_d_-values of the membranes themselves range between 0.027 and 0.20 m. All tested adhesive joints are considerably more vapour tight than the Norwegian recommended value for breather membranes (<0.5 m). However, the mould growth analysis shows that the risk of mould growth is low in most practical cases, except when using adhesive joints with the highest vapour resistance in roofs assembled during autumn.

## 1. Introduction

In recent years, climate change and environmental impact have gained increasing attention, also in the building sector. Roughly 40% of energy consumption in Europe is used in construction and operation of buildings [1]. National building authorities strengthen requirements for energy efficiency by limiting air leakages and thermal transmission in order to reduce energy consumption [2]. Additionally, the changing climate causes warmer and wetter weather and increased frequency of storms and extreme weather [3,4]. As a result, buildings must be robustly constructed to withstand the consequences of changing climate. In the Nordic countries, precipitation, temperature, and humidity varies greatly from coastal to continental climate and throughout the seasons. Coastal areas are commonly wetter and have temperatures moderated by the Gulf stream, while continental areas are dryer and experience colder average temperatures in the winter months [5]. These weather conditions cause significant strain on Norwegian building envelopes [6].

Roofs in Norway are commonly constructed as pitched wooden roofs with a ventilated air cavity [7]. The air cavity provides ventilation by airflow beneath the roofing, which transfers moisture from the roof structure to prevent condensation and mould growth. The roof tiles shield the underlay roof from most of the precipitation load, while any moisture that passes the roof tiles is allowed to dry due to the ventilated air cavity. This design principle is referred to as dual barrier weatherproofing or “two-stage weatherproofing” [8]. The inner surface of this air cavity is the underlay roof, which may serve as the second barrier of the weatherproofing. The underlay roof needs to be able to withstand some moisture load and allow drying. There also needs to be a wind barrier layer, to prevent wind-driven convection from cooling the roof insulation. The dual functions of moisture protection and wind barrier are often combined in a single product, a combined wind barrier and underlay roof. This product provides more efficient construction and less materials than two separate layers. The nomenclature of this type of product remains somewhat poorly defined, with several different names in use. The authors are aware of the following names: “Combined wind barrier and underlay roof” (translation of Norwegian term) [9], “Breathable membrane” and “Diffusion-open layer” (SIGA) [10], “Underlay membrane and roof protection film” (Würth) [11], “Breathable roof underlays” (Masterplast) [12], “Vapour permeable underlay” and “Pitched roof breather membrane” (BMI) [13], and “Breather membrane” (DuPont) [14]. Technical differences between these terms may exist, but the authors have not been able to reveal them. For its brevity, the term “Breather membrane” will be used in this article.

The purpose of breather membranes is to prevent rainwater and wind from entering the building envelope [15]. It is a board or membrane layer beneath the roofing that must be vapour permeable to permit moisture to diffuse through to the outside. To achieve this property, the layer should, according to Norwegian recommendations, have a water vapour resistance (s_d_-value) of less than 0.5 m [9]. There must also be sufficient ventilation of the air cavity to ensure transport of humid air.

**Figure 1:**
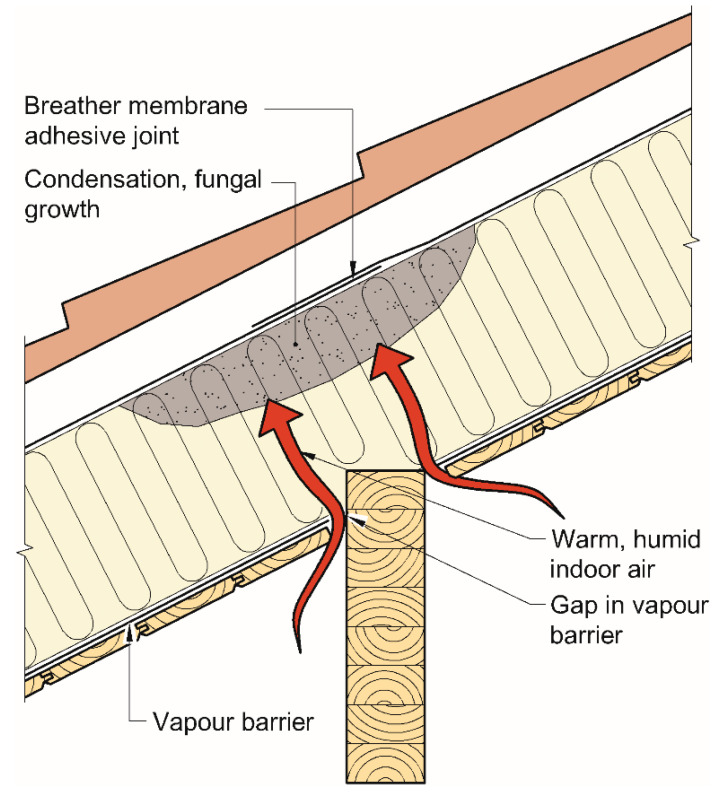
Accumulation of humidity causing condensation and risk of mould growth. Figure adapted from [21].

Several materials exist with the desired properties to allow moisture protection and vapour diffusion. However, some concern has been raised about the joints between membrane sheets. Tape and adhesive joints are frequently used as a method to ensure air and water tightness [16]. Previous research suggest that adhesive joints are the most airtight solution for timber buildings [17]. Sheets of roof underlay are commonly joined together by a strip of adhesive material running along the edge of the sheets. The vapour permeability of adhesive strips may be significantly lower than that of the rest of the underlay [18,19]. Moisture accumulating underneath these joints may be prevented from drying out, causing moisture damage to the building envelope. The concern is illustrated in Figure 1, where humid air leaking from the interior side condensates and moisture accumulates beneath the joints of the combined wind barrier and underlay roof. The most common sources of moisture are air leakages from the inside and built-in moisture. If the structure cannot dry adequately, there is a great risk of mould growth [15,20].

Scientific literature on the moisture properties of adhesive building membrane joints has not been identified by the authors despite extensive searching. This deficiency was also reported in a previous study on construction tape [18]. Identified literature on adhesives in the building industry seems to be primarily concerned with mechanical properties such as bonding strength [22,23], the long-time performance and durability of adhesives [16,24], or airtightness [17]. These topics are all outside the scope of the present study.

A Norwegian study has investigated building defects of pitched roofs reported between 1993 and 2002 to gain an overview of the main causes of roof defects [25]. A total of 67% of the reported roof defects were found to be caused by precipitation and condensation of indoor air. A follow-up study with data from 2017–2020 found that 71% of all reported building defects were related to moisture [26]. Precipitation moisture was found to be the most common cause of defects, comprising 41% of the reported defects. As the climatic loads are expected to increase [3], it is ever more essential that the roof remains capable of withstanding precipitation. For breather membranes, the weakest points are the joints between sheets, creating the biggest risk of air and moisture leaks in these locations [15]. A total of 9% of the defects were caused by moisture condensing from leaks of indoor air, making it the second largest cause of moisture-related defects [26]. The number is somewhat lower than it was in the 1993–2002 data, due to stricter requirements of airtightness in buildings.

It is estimated that 2–6% of the costs of a construction project are related to the repair of defects [25,27,28]. Repairs of moisture defects are also associated with significant CO_2_ emissions due to the large amount of energy needed to dry the building [29]. It therefore remains important to further reduce the extent of roof defects through understanding of the moisture properties of building materials. Breather membranes constitute an important component determining the roof’s ability to shed moisture. It is unknown how the drying ability is impacted by the adhesive joints, which may be considerably more vapour-tight than the rest of the membrane and thus might form “pockets” where moisture is not able to dry out. Considering these general concerns, this article seeks to answer the following research questions:

How can the water vapour resistance of the adhesive joints of breather membranes be determined?

What is the water vapour resistance of the adhesive joints of commercially available breather membranes?

What are the implications of the water vapour resistance of these joints, with regards to the drying capacity of roof structures?

The measurement method of NS-EN ISO 12572:2016 [30] is used to determine the water vapour permeability of adhesive joints under standardized stable conditions, to create a comparison between available products. The effects of variable conditions, i.e., moisture and temperature, on product properties are considered beyond the scope of the present study.

The primary novelty of the present study is to examine relevant moisture properties of adhesive joints and determine whether they are cause for concern about mould growth and wood decay in several Norwegian climates. Due to the extensive use of wood as a building material for roofs in the Nordic countries, this knowledge is essential to the design of climate-adapted buildings.

## 2. Materials and Methods

### 2.1. Tested Materials

A number of products have been granted Technical Approval for use in the Norwegian building sector by SINTEF Certification. This form of certification ensures that the product is suitable for use in the Norwegian climate and that it conforms to Norwegian regulations [31]. A list of certified products was found at [32]. Specimens of these products were acquired from a commercial building material supplier. Some specimens were produced from leftover materials from the certification tests; this ensured that the material properties of the specimen in question had been thoroughly documented ahead of the testing. Table 1 lists the tested products and their properties, where brand names have been anonymized.

The detailed material composition of the breather membrane products is not public information. Product datasheets describe the products as consisting of spun or woven polyester with a microporous membrane in the middle or covering one surface. The composition of the joint adhesive is not described in datasheets, except a brief mention of “acryl-based tape” in the Technical Approval certificate for product J.

The products must classify as breather membranes and include adhesive joints. All available products that fulfil these qualifications were tested to obtain a broad perspective of the water vapour resistance in adhesive joints. In total, adhesive joints of eleven products were examined according to NS-EN ISO 12572:2016, commonly known as the cup method [30].

### 2.2. Test Sample Preparation

Breather membrane sheets feature adhesive strips along each edge, on opposite sides of the membrane. These adhesive strips were cut off the sheets and into slim ribbons approximately 200 mm long. To ensure straight, clean edges, a cutting machine was used. The blade was placed slightly within the adhesive area, making sure the entire ribbon was covered with adhesive. Consequently, the width of the ribbons was somewhat narrower than the adhesive strip width listed in Table 1.

**Figure 2:**
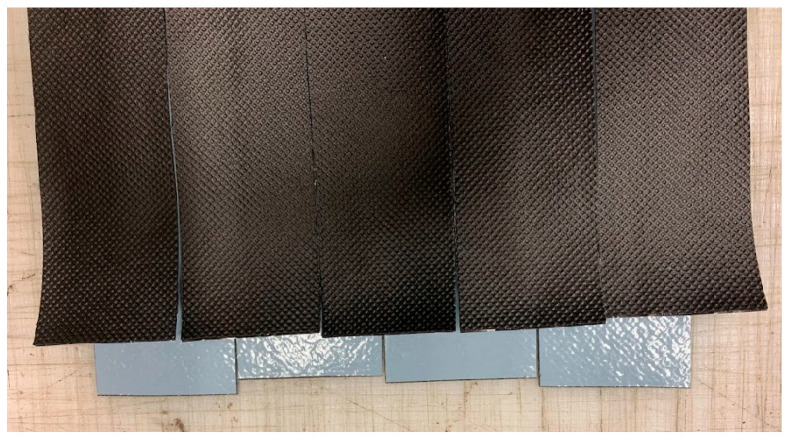
Visualisation of overlapping bands in a test sample. The picture was taken before the samples were glued together and circular samples of 174 mm were stamped out.

The ribbons were glued together parallelly, demonstrated in Figure 2, to form a composite sheet consisting only of adhesive strips. Adhesive strips were joined together according to the manufacturers’ assembly instructions. Joints were overlapping by half a ribbon’s width, at a minimum of 5 mm, to reduce the risk of leakages through a sample. Circular samples with a diameter of 174 mm were cut out of the composite sheets. The number of joints within a sample depends on the width of the adhesive strip on the product in question. For each product, five samples were tested.

The prepared samples were stored in the test chamber for conditioning for two days before being mounted into sample containers. The test chamber was kept stable within the requirements of NS-EN ISO 12572:2016, i.e., temperature (23 ± 5) °C and a relative humidity (RH) of (50 ± 5)% [30].

Before the sample containers were mounted, the thickness of each sample was measured using callipers. The thickness was measured at five random points on the sample, and the reported thickness is the average of all measurements for a series of samples. Several of the products contain a reinforcing mesh, making the thickness variable and difficult to measure.

**Figure 3:**
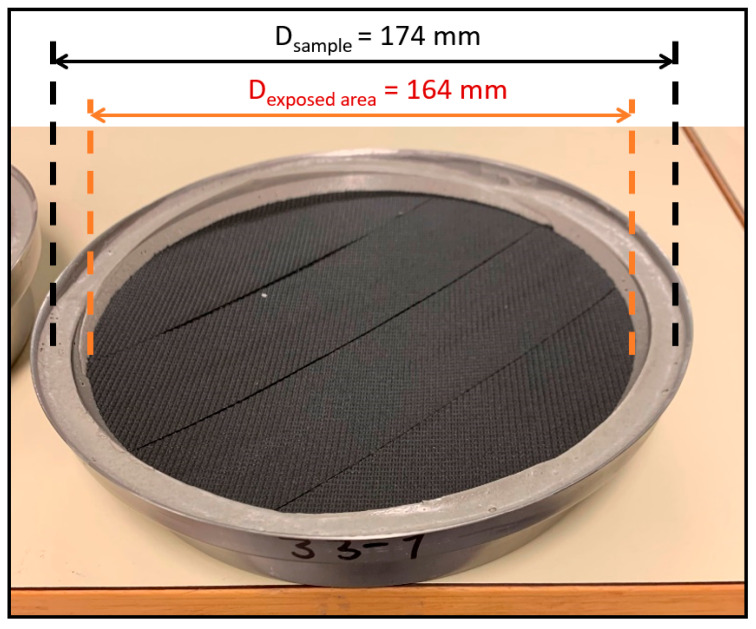
Prepared sample container, with the diameters of the sample and of its exposed area illustrated. The sample is circular.

A sample container consists of a cup, a saline solution, the sample, and a sealant. The cup was filled with a saturated solution of potassium nitrate, KNO_3_, up to 15 mm below the sample. The sample was mounted centrally over the cup, with the back side facing the saline solution. A metal ring was used to hold the sealant in place while it hardened. The sealant consisted of 300 g modelling clay and 60 g beeswax, which was carefully heated and poured between the metal ring and the edge of the cup. When the sealant had hardened, the metal ring was removed and the sample container was ready, as shown in Figure 3.

### 2.3. Test Procedure

The sample containers were weighed using a precision scale inside the test chamber [30]. The scale has an accuracy of 0.001 g and was protected from air currents inside a glass box. Before and after each measurement series, the scale was calibrated using a 1000 g control weight. In addition, climate data for the period between measurements were registered. The average temperature, RH, and barometric pressure in the chamber were logged in a spreadsheet along with the measured weight of the samples. The spreadsheet calculated the water vapour permeability from the measured weight change of the sample container. If five consecutive measurements yielded a calculated permeability within ±5% of the same value, the sample was considered stable, and permeability could be determined using these values.

The time interval between each measurement was dependent on the assumed vapour permeability of the product (which may be declared by the manufacturer, estimated from the characteristics of the sample, or the approximate value will become apparent after a few measurements). Table 2 shows the relation between vapour permeability and the time interval between measurements, as determined by SINTEF’s internal measurement procedures.

### 2.4. Simulation of Moisture Transport and Mould Growth

To evaluate the impact of vapour-resistant adhesive joints, moisture transport was simulated in WUFI^®^ 2D (version 4.3), a building physics simulation software by Fraunhofer IBP in Holzkirchen, Germany. The simulation software includes thermal conduction and vapour diffusion. Convective transport of vapour and heat was disregarded. The aim of these calculations was to investigate whether vapour-resistant adhesive joints prevent the drying of roof assemblies. RH and temperature data were analysed for mould growth risk in the add-on program WUFI Mould Index VTT.

Certain input values remained unchanged throughout the calculations. The climatic parameters were selected for a worst-case scenario for moisture. The location of the simulated setup was hence chosen to be Kristiansund, a coastal town in north-western Norway, which experiences mild winters, cool summers, and frequent rain throughout the year. The roof was angled at 30°, facing north. The input parameters for the simulations are listed in Table 3. The combinations of parameter variants used in each simulation are listed in Tables 6 and 7.

**Figure 4:**
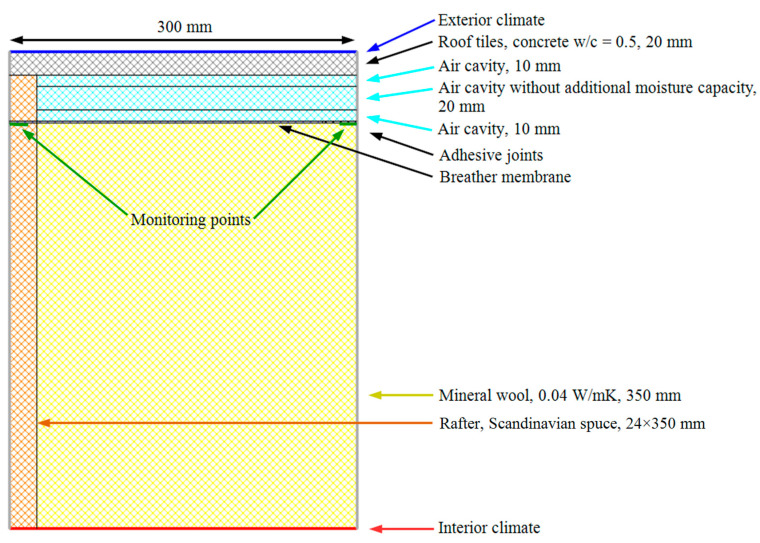
Roof geometry simulated in WUFI 2D.

The model of the roof assembly is illustrated in Figure 4. It was identical for all simulations, except for the placement of the adhesive joint. The model was symmetrical along the adiabatic border. The rafters were 48 mm × 350 mm structural beams of Scandinavian spruce. The chosen thickness in combination with structural timber caused a high level of moisture compared to other available concepts but is still a commonly built roof assembly. The roof insulation consisted of mineral wool between the rafters. A breather membrane was mounted as an underlay roof across the rafters, along the top of the mineral wool layer. The adhesive joint was laid parallel to the rafters, either directly atop the rafters or above the mineral wool layer. Rolled membrane sheets are usually laid perpendicular to the rafters but can also be laid in parallel. In this study, the parallel direction was chosen because it creates the most conservative situation, while also being feasible to simulate in WUFI 2D. The name and properties of materials used in simulations are listed in Table 4.

The setup included a ventilated air cavity and roof tiles. The air cavity was divided into three layers, where the inner- and outermost layers have a higher moisture capacity than the middle layer, to create a more realistic approximation of the drainage and drying effect of the air cavity.

On the interior side, the assembly ended with a transition layer to the interior climate. The vapour barrier, thermally insulated battening, and interior cladding were excluded to reduce the computation time. This simplification does not affect drying in the exterior region of the assembly.

The examined output of the simulations was hourly data for temperature and relative humidity at the monitoring point, which was moved between the two positions shown in Figure 4 depending on the location of the adhesive joint (above rafter or above insulation). The monitoring point was always located close to the adiabatic border, right below the adhesive joint.

The results of the WUFI 2D simulation were controlled for mould growth risk, using the accessory plugin WUFI Mould Index VTT. The plugin is based on a mould growth model described by Viitanen et al. [33,34], outputting a mould growth index from 0 to 6. The model takes into account the relative humidity, temperature, time, and type of material. The index is annotated using a “traffic light model”, where a green label means acceptable risk, yellow means the solution should be evaluated further, and red means additional measures are necessary. The requirements for the different colours depend on which part of the structure is analysed. For materials not in direct contact with interior air, a mould growth index of 3 should not be exceeded. This requirement is relevant for the assessment of mould growth under the adhesive joints of breather membranes. The threshold between the green and yellow level was set to be 2.0.

## 3. Results

The results from the laboratory measurements are presented in Table 5, alongside the measured and declared vapour permeability of the membrane itself. The products are annotated A to K for the purposes of anonymization. The column furthest to the right lists the standard deviations of each measurement series. Note that measurement series B and B* represent the same product, with samples produced from the same sheet specimen, measured a few months apart. The same is true of series I and I*. The difference in measured values between the series highlights the challenge of measuring the material properties of some products. Product B was found to be particularly troublesome, with s_d_-values stabilizing anywhere between 3.4 and 15 m for different samples prepared from the same specimen. It is conjectured that the adhesive of this product may have been applied unevenly during the manufacturing process, which could explain the large discrepancies. Table 6 and Table 7 present the parameters of the WUFI simulations and show the mould growth index for each simulation case in the bottom row. Note that Table 7 is a continuation of Table 6.

## 4. Discussion

### 4.1. Determination of Water Vapour Resistance

A trend observed through the laboratory measurements is that the products behave quite differently and exhibit various degrees of vapour permeability. Some products reach a stable change in weight after a short time and only require the minimum number of measurements before an s_d_-value can be determined. Others appear to become more vapour permeable as time goes on and need several months before the permeability stabilizes. None of the sample containers show signs of salt efflorescence or other visible defects that could have compromised the measurements.

Two of the eleven tested products were measured twice. Product B exhibited very inconsistent properties (large standard deviation) in the first round of measurements and was re-tested in an attempt to verify its vapour permeability with greater accuracy. Product I was re-tested alongside it to verify the consistency of the methodology. As seen in Table 5, the series B and B* exhibit very large standard deviations and different mean values, so, even though the measurement series behave alike, it is not possible to accurately determine a vapour permeability for product B. Product I, which was also tested again, exhibited a much more consistent behaviour with comparable results between the two measurement series. As such, the issue appears to lie with product B and not the measurement procedure. However, it remains unknown why the vapour resistance of product I has increased by 10% (from 10 to 11 m) between measurements, since the samples were created from the same sheet of membrane product. The time difference of 4 months between measurements may be conjectured to have some influence, but this remains untested.

The ribbons made from the adhesive strips of the various products are mounted parallelly with an overlap of roughly half a ribbon’s width to ensure that vapour does not leak through the joints but diffuses through the material. Product J features the narrowest adhesive strip at 30 mm, thus having the most ribbons and so the most joints per sample out of all the products. Even so, the vapour resistance of product J is one of the highest among all the tested products (s_d_-value of 18 m). Conversely, product E is the most vapour permeable (s_d_-value of 1.1 m), yet it has the widest adhesive strips (75 mm) and hence the fewest joints per sample among the products. These examples show that there is no clear correlation between the number of joints per sample and the amount of vapour permeating through. It can also be inferred that the method for creating the samples adequately maintains sample integrity. The adhesive covers the entire surface of the sample ribbons, the overlap in the joints is large, and the ribbons are laid together against a flat surface, which should result in airtight joints. Measurement results appear to indicate that satisfactory airtightness was achieved.

### 4.2. Measured Water Vapour Resistance

The breather membranes exhibit a large variation in the vapour resistance of their adhesive joints. None of the tested products can be considered vapour open, as their s_d_-value is greater than 0.5 m. The most vapour open adhesive joints have an s_d_-value of 1.1 m, while the most vapour tight is 32 m. Many of the products exhibit quite low vapour resistance, between 1 and 3 m. These products can be considered unproblematic in terms of moisture and mould growth, as the adhesive joints comprise a very small portion of the roof surface. The vapour resistance of 32 m is presumably an extreme value for products in this segment, but it may prove unfortunate if several conditions coincide to facilitate mould growth.

There appears to be no correlation between the vapour resistance of the membrane and the adhesive joints, as illustrated by product E, which has the highest s_d_-value out of all the membrane sheets but the lowest among the adhesive joints. No correlation has been observed between the thickness of the samples and their vapour resistance as well. At 2.10 mm on average, product B makes the thickest samples but has a very inconsistent s_d_-value, between 3.4 and 15 m. Product A has the second thickest samples (1.83 mm) and the highest s_d_-value at 32 m but is barely thicker than the most vapour-open product, E, whose samples are 1.80 mm thick on average.

### 4.3. Implications

Simulations in WUFI 2D and WUFI Mould Index VTT show that several conditions must be fulfilled before vapour-tight adhesive joints may cause any significant mould growth. Through 30 simulations, multiple parameters were investigated to determine their significance for mould growth risk in a roof assembly featuring vapour-tight adhesive joints.

The most vapour-tight adhesive joint (s_d_-value 32 m) was chosen as the baseline value, with the second and third least permeable products (s_d_-values 18 and 11 m) used as variations. These were the three least permeable products found in the laboratory investigation and are not representative for all products on the market but were chosen to test the most critical situation. Even so, no combination of conditions was found to result in mould growth when the adhesive joint was placed over the insulation layer. With the joint placed along the rafters, moisture and vapour may be prevented from diffusing through the breather membrane, which may result in mould growth. It is also seen that the time at which the roof is completed (and, hence, the transport of built-in moisture begins) plays a significant role for mould growth. Every simulation that begins in April yields low levels of mould growth risk, as the warm spring weather causes the structure to dry out relatively fast. Conversely, a roof completed in autumn faces higher risk. Of the four climate locations tested, Kristiansund was the least favourable, followed by Trondheim. In these locations, the most critical solution yielded unacceptable mould risk for all three types of adhesive joints. In Oslo, only the least permeable joint created issues, while in Tromsø none of the solutions were considered unacceptable. Note that the calculations deliberately consider structural timber (rafters) of relatively large volume, thus containing a relatively large volume of built-in moisture. For instance, using wooden laminate I-beams (of less volume) instead of structural timber would drastically lower the risk. Calculations 20–23 also show that the risk is reduced if the initial wood moisture content is lowered from 20 weight-% to more sensible levels of 18 or 15%. The simulations also consider cases where the volume of wood in the assembly is doubled, by lowering the spacing of the rafters from 600 to 300 mm. This solution is uncommon but may be chosen in cases of long spans or around chimneys, windows, or other perforations of the roof assembly. This solution increases the moisture risk but only to unacceptable levels when combined with high initial moisture content in the wood materials. At initial moisture levels of 15 weight-%, the combination of vapour-tight adhesive joints and high wood volume is not problematic.

Note also that the adhesive joints in the simulations were mounted parallel to the rafters, a different direction than what is usually the case. Assembly instructions recommend placing the joints perpendicular to the rafters instead. The chosen setup makes the adhesive joints cover a much larger portion of the rafters than is usually the case. This solution was chosen to make simulations more feasible and to investigate a more critical case. It is assumed that a conventional mounting of the adhesive joints will not lead to high risk of mould growth.

All told, for a well-built roof, the vapour permeability of adhesive joints does not appear to be critical for mould risk. Only when several unfortunate factors are combined may significant mould risk occur.

However, the simulations do not consider defects, moisture leaking into the structure from humid indoor air, or rainwater intrusion. If moisture is periodically allowed to enter the roof assembly, mould growth may occur where vapour diffusion is prevented. Flaws, defects, or damage to the roof or the interior vapour barrier may all cause significant moisture intrusion. With vapour-tight adhesive joints, the assembly may be at risk of mould growth if defects or leaks occur.

This article has only considered the standardised determination of material properties in laboratory conditions according to NS-EN ISO 12572:2016. However, a study of breather membranes in real winter conditions [35] found that, for most products, the vapour resistance was about the same at freezing temperature as at room temperature. Only one product saw a significant increase in vapour resistance at freezing temperature.

## 5. Conclusions

This article has presented an assessment of the water vapour resistance of adhesive joints of breather membranes used in ventilated roofs. The consistency of the laboratory results indicates that creating samples from adhesive strips of material is a sufficiently reliable way to measure water vapour resistance of these joints.

The study has found that the adhesive joints of commercially available breather membranes exhibit a water vapour resistance ranging from 1.1 to 32 m, much higher than what is recommended for membrane products (<0.5 m). However, the adhesive joints comprise only a very small portion of the total roof area. The membranes themselves were measured to have s_d_-values ranging between 0.027 and 0.2 m. Computer simulations indicate that the risk of mould growth associated with the low permeability of adhesive joints is very low, and that a multitude of unfortunate factors need to coincide for significant mould growth to occur.

Future work on the topic should aim to validate the simulation results by investigating the performance of adhesive joints in full-scale tests, either in the field or in a laboratory setting. It remains to be seen how varying weather with different and fluctuating levels of moisture and temperature may affect the properties of the materials in the long term. Unconventional joint solutions and geometries may also be investigated, for instance, complex roofs where rafters and barrier layers may interact with windows, chimneys, or other perforations of the roof assembly.

## Figures and Tables

**Table 1:** Overview of Tested Products (Brand Names Have Been Anonymized).

Product Identification	Width of Adhesive Area (mm)	Specimen Thickness (mm)
A	60	1.83
B	50	2.10
C	40	1.32
D	45	1.63
E	75	1.80
F	90 top side/45 back side	1.33
G	50	1.57
H	50	1.61
I	70	1.34
J	30	1.58
K	50	0.84

**Table 2:** Measuring Intervals Depending on the Vapour Permeability of the Product.

s_d_-value	<0.5 m	0.5–2 m	2–10 m	>10 m	>50 m
Measuring interval	Morning and afternoon	1 day	2–7 days	7–14 days	14–28 days

**Table 3:** Input Parameters for the Simulations in WUFI 2D and WUFI Mould Index VTT.

WUFI 2D Settings	Standard Parameters	Variations
Start date	1 September	1 April
Number of Time Steps	8760	
s_d_-value	32 m	18 m, 11 m
Placement of adhesive joint	Above rafter	Above insulation
Built-in moisture	20%	18%, 15%
Distance between rafters (c/c)	600 mm	300 mm
Exterior climate	Kristiansund	Oslo, Tromsø, Trondheim
Orientation	North	
Inclination	30°	
Short-Wave Radiation Absorptivity	0.8	
Indoor Climate Temperature	20 °C	
Indoor Relative Humidity	Humidity Class 2	
**WUFI Mould Index settings**		
Sensitivity Class	Sensitive	
Material Class	Relevant decline	
Type of Surface	Planed	
Type of Wood	Softwood	
Occupant exposition class	Surfaces inside constructions without direct contact to indoor air	

**Table 4:** Material Properties from WUFI 2D.

Materials	Density (kg/m^3^)	Heat Conductivity (W/mK)	S_d_-Value (m)
Scandinavian spruce transverse direction II	390	0.13	38
Mineral Wool	60	0.040	0.46
Breather membrane	210	2.3	0.099
Adhesive joint	210	2.3	32, 18, 11

**Table 5:** Results From Laboratory Measurements Compared to Declared and Measured Water Vapour Resistance of Breather Membranes. Measured S_d_-Value of Breather Membranes Are Collected From SINTEF Archives. Declarations of Performance Are Gathered From SINTEF Technical Approval or The Product Datasheets. Test Series Marked with an Asterisk (*) are the Results from Preliminary Studies.

Product Identification	Measured S_d_-Value of Breather Membrane (m)	S_d_-Value from Declaration of Performance (m)	Measured S_d_-Value of Adhesive Joints (m)	Standard Deviation of the Mean S_d_-Value of Adhesive Joints (m)
A	0.18	≤0.14	32	0.64
B	0.073	≤0.08	8.1	1.7
B *	0.073	≤0.08	7.5	2.1
C	-	≤0.06	2.9	0.33
D	-	0.08 − 0.02/+0.10	1.5	0.024
E	0.20	0.08 (0.05–0.19)	1.1	0.014
F	0.027	0.03	3.7	0.18
G	0.10	≤0.10	4.8	0.5
H	-	0.03 ± 0.02	1.7	0.05
I	0.077	0.06 ± 0.01	11	0.09
I *	0.077	0.06 ± 0.01	10	0.12
J	0.12	0.12	18	0.17
K	0.049	0.03 ± 0.02	1.7	0.015

**Table 6:** Parameter Study and Results from Simulation in WUFI 2D and WUFI Mould Index VTT. Calculations 1–15.

	1	2	3	4	5	6	7	8	9	10	11	12	13	14	15
Adhesive joint above rafter	X	X			X	X			X	X	X	X	X	X	X
Adhesive joint above insulation			X	X			X	X							
Start date: 1 September	X		X		X		X		X		X	X	X	X	X
Start date: 1 April		X		X		X		X		X					
Vapour resistance adhesive joints: 32 m	X	X	X	X							X			X	
Vapour resistance adhesive joints: 18 m					X	X	X	X				X			X
Vapour resistance adhesive joints: 11 m									X	X			X		
Exterior Climate: Kristiansund	X	X	X	X	X	X	X	X	X	X					
Exterior Climate: Oslo											X	X	X		
Exterior Climate: Tromsø														X	X
Exterior Climate: Trondheim															
Built-in Moisture: 20%	X	X	X	X	X	X	X	X	X	X	X	X	X	X	X
Built-in Moisture: 18%															
Built-in Moisture: 15%															
c/c: 600 mm	X	X	X	X	X	X	X	X	X	X	X	X	X	X	X
c/c: 300 mm															
**Mould Index:**	3.4	1.7	0.5	0.1	3.2	1.6	0.4	0.1	2.5	1.5	2.1	1.9	1.4	1.9	1.8

**Table 7:** Parameter Study and Result from Simulation in WUFI 2D and WUFI Mould Index VTT. Calculations 16–30.

	16	17	18	19	20	21	22	23	24	25	26	27	28	29	30
Adhesive joint above rafter	X	X	X	X	X	X	X	X	X	X	X	X	X	X	X
Adhesive joint above insulation															
Start date: 1 September	X	X	X	X	X	X	X	X	X	X	X	X	X	X	X
Start date: 1 April															
Vapour resistance adhesive joints: 32 m		X			X	X			X			X			X
Vapour resistance adhesive joints: 18 m			X				X	X		X			X		
Vapour resistance adhesive joints: 11 m	X			X							X			X	
Exterior Climate: Kristiansund					X	X	X	X	X	X	X	X	X	X	X
Exterior Climate: Oslo															
Exterior Climate: Tromsø	X														
Exterior Climate: Trondheim		X	X	X											
Built-in Moisture: 20%	X	X	X	X					X	X	X				
Built-in Moisture: 18%					X		X					X	X	X	
Built-in Moisture: 15%						X		X							X
c/c: 600 mm	X	X	X	X	X	X	X	X							
c/c: 300 mm									X	X	X	X	X	X	X
**Mould Index**	1.4	3.1	2.9	2.5	2.1	0.9	1.9	0.8	4.3	4.2	3.7	3.1	2.9	2.3	1.6

## Data Availability

Not applicable.
